# The effect of HPV vaccination on precancerous lesions and invasive cervical cancer in a low-vaccination-coverage setting in Germany: a retrospective cohort analysis

**DOI:** 10.1016/j.lanepe.2026.101830

**Published:** 2026-08-26

**Authors:** Gifty Baffour Awuah, Sandra Fett, Gunther Schauberger, Vanesa Osmani, Martin Tauscher, Marianne Röbl-Mathieu, Marion Kiechle, Stefanie J. Klug

**Affiliations:** a Chair of Epidemiology, Technical University of Munich, TUM School of Medicine and Health, Munich, Germany; b The Bavarian Association of Statutory Health Insurance Physicians (KVB), Munich, Germany; c Technical University of Munich, TUM School of Medicine and Health, Department of Obstetrics and Gynaecology, Munich, Germany; d Gynaecologist's Office, Adams-Lehmann-Str. 36, Munich, Germany

**Keywords:** HPV, Vaccination, Cervical cancer, Precancerous lesions, Germany

## Abstract

**Background:**

HPV vaccination coverage among 18-year-old women in Germany was 61% in 2024, despite the recommendation for girls since 2007. We assessed the association between HPV vaccination and risk of precancerous lesions and invasive cervical cancer in Bavaria, Germany's largest state.

**Methods:**

We conducted a retrospective cohort analysis of females born 1990–2005, covering 17 years of follow-up (2008–2024), using health claims and prescription data from the Bavarian Association of Statutory Health Insurance Physicians (KVB). Cumulative incidence of precancerous cervical lesions and invasive cervical cancer was estimated using the Kaplan–Meier method, and risks were estimated using Cox regression models. Analyses accounted for factors such as HPV vaccination status, age at vaccination, pre-vaccination contraceptive prescription, cervical cancer screening participation, and place of residence.

**Findings:**

Among 669,053 females, 33.76% (225,899/669,053) were fully vaccinated, 13.34% (89,268/669,053) partially vaccinated, and 52.89% (353,886/669,053) unvaccinated. 8965 precancerous lesions and 669 invasive cervical cancers were diagnosed in females aged 19–34 years. By age 34 years, cumulative incidence of precancerous lesions and invasive cervical cancer was 0.025 (95% CI: 0.020, 0.029) and 0.001 (95% CI: 0.001, 0.002) in fully vaccinated cohorts, and 0.041 (95% CI: 0.040, 0.043) and 0.004 (95% CI: 0.003, 0.004) in unvaccinated cohorts, respectively. Adjusted hazard ratios among fully vaccinated females were 0.48 (95% CI: 0.45, 0.52) for precancerous lesions and 0.35 (95% CI: 0.26, 0.47) for invasive cervical cancer.

**Interpretation:**

HPV vaccination is associated with a lower incidence of precancerous lesions and invasive cervical cancer. This is the first direct evidence of the effectiveness of the HPV vaccine in Germany.

**Funding:**

No external funding.

**Research in context**

**Evidence before this study**

Randomised controlled trials have demonstrated the efficacy of the HPV vaccine against precancerous lesions and invasive cervical cancer. We searched PubMed for real-world studies of HPV vaccine effectiveness against invasive cervical cancer published between January 2015 and March 2026, using the terms (HPV vaccination) AND (cervical cancer) AND (effectiveness) AND (real-world). We identified cohort studies with individual-level vaccination data from Sweden, Denmark, the Netherlands, and Scotland that reported substantial reductions in cervical cancer risk among vaccinated individuals. Unlike Germany, Sweden, Denmark, and Scotland have organised, mostly school-based vaccination programmes and higher vaccination coverage of 80–90%.

**Added value of this study**

This is the first study to evaluate the real-world effectiveness of HPV vaccination in a country without an organised HPV vaccination programme and with relatively low HPV vaccination rates. We used health claims data from Bavaria, Germany's largest federal state, with a population of about 13 million, and analysed almost 700,000 females with information on individual vaccine status and a long follow-up period of 17 years. Only 47% of the females received at least one HPV vaccination and only 34% were fully vaccinated. The cumulative incidence of precancerous cervical lesions and invasive cervical cancer was lower in fully vaccinated cohorts compared to their unvaccinated counterparts. HPV vaccination was associated with substantial reductions in disease risk, with fully vaccinated females showing a 52% lower risk of precancerous lesions and a 65% lower risk of invasive cervical cancer compared with unvaccinated females. Reduction in disease risk was slightly lower among partially vaccinated females and among those vaccinated only once. Hormonal contraceptive prescriptions before vaccination were associated with a statistically significantly increased risk of diagnosis for both outcomes.

**Implications of all the available evidence**

Real-world evidence from countries with high HPV vaccination coverage, such as Sweden, Denmark, and Scotland, shows high effectiveness of HPV vaccination to reduce cervical cancer. Compared with countries with higher vaccination coverage, the reduction observed in Germany, a country with lower HPV vaccination coverage, was lower. Measures to increase HPV vaccination are needed in countries with lower coverage to reduce the HPV disease burden and ultimately eliminate invasive cervical cancer according to the WHO cervical cancer elimination goals.

## Introduction

Cervical cancer is the second most common gynaecological malignancy globally, with 662,301 new cases and 348,874 deaths recorded in 2022.^1^ In Germany, it is the fourth most common gynaecological cancer, with 4300 new cases and 1413 deaths recorded in 2023.^2^ Nearly all cases of cervical cancer are caused by high-risk human papillomaviruses (HPV).^3^ The first HPV vaccine was licensed in 2006 to prevent HPV infections and ultimately contribute to the elimination of cervical cancer.^4^ HPV vaccination is a key pillar of the World Health Organisation (WHO) cervical cancer elimination strategy, which aims to vaccinate 90% of all girls globally by 2030.^4^

The WHO^5^ recommends HPV vaccination at ages 9–14, while the American Advisory Committee on Immunisation Practices (ACIP)^6^ recommends vaccination at 11–12 years, although vaccination can be started at 9 years. In Germany, the German Standing Committee on Vaccination (STIKO) first recommended HPV vaccination in 2007 for all girls aged 12–17.^7^ This was updated in 2014, to vaccination at 9–14 years, with a possibility of catch-up vaccination until the age of 18,^8^ and extended to boys in 2018.^9^ Vaccination costs are fully covered by statutory health insurance for eligible groups, however, some insurance companies also refund vaccination costs for older age groups. Unlike countries like Australia,^10^ Denmark,^11^ Sweden,^12^ and Scotland,^13^ which have organised, mostly school-based vaccination programmes with coverage rates around 80–90%,^14^ HPV vaccination in Germany is opportunistic. Parents must present their children to an office-based physician (e.g. paediatrician, general practitioner, or gynaecologist) to receive HPV vaccination. Consequently, vaccination coverage in Germany has only slowly increased from 27% in 2011 to 55% in 2024 among 15-year-old girls and from 46% in 2014 to 61% in 2024 among 18-year-olds.^15^^,^^16^ In Germany, cervical cancer screening, like HPV vaccination, was opportunistic until 2020, offering annual Pap smears from age 20 years.^17^ Since 2020, women aged ≥35 years receive HPV and Pap co-testing every 3 years, while those aged 20–34 years continue to receive annual Pap smears.^18^ In 2016, the annual cervical screening participation rate ranged from 58% to 68% among 20–35-year-olds.^19^

Although randomised controlled trials have demonstrated the efficacy of the HPV vaccine against precancerous lesions and invasive cervical cancer,^20^ real-world effectiveness against invasive cervical cancer has only been reported in few cohort studies from Sweden, Denmark, the Netherlands, and Scotland.^21^, ^22^, ^23^, ^24^ Prior studies in Germany have focused on anogenital warts and precancerous lesions.^25^^,^^26^ A recent age-period-cohort analysis of German cancer registry data showed lower cervical cancer incidence in birth cohorts born during the HPV vaccination era, suggesting a vaccination effect.^27^ However, because vaccination status was unavailable, this study provided only an indirect estimate of the vaccination impact.

Our study aims to assess the effect of HPV vaccination on precancerous lesions and invasive cervical cancer in Germany by analysing a population-based cohort of females born between 1990 and 2005. We utilise data from the Bavarian Association of Statutory Health Insurance Physicians (KVB), covering approximately 11 million of the 13 million inhabitants of Bavaria, Germany's largest state. This analysis generates the first direct evidence of the impact of HPV vaccination on cervical cancer in Germany, a country without an organised HPV vaccination program and with low HPV vaccination rates.

## Methods

### Study design and population

Using health claims and prescription data from KVB, we performed a retrospective cohort analysis on all females born between 1990 (the oldest birth year with available data on vaccination) and 2005 (aged 19–34 years in 2024) residing in Bavaria between 2008 and 2024. Females without a permanent residence in Bavaria were excluded because HPV vaccination received before or after migration could not be ascertained. Although the STIKO recommendation for HPV vaccination began in 2007, data were available only from 2008. The girls or women entered the cohort starting from the recommended vaccination age of 9 years or from 1st January 2008, whichever came later. All eligible females were followed until censoring, until a diagnosis of any lesions (precancerous cervical lesion or invasive cervical cancer), or until 31st December 2024, whichever came first. Censoring occurred at the last entry of the person in the KVB database, where possible reasons for this could be emigration from Bavaria, death, or transition to a private insurance scheme. Participants diagnosed with precancerous lesions remained under follow-up for invasive cervical cancer.

### Data source

The KVB database covers all statutorily insured individuals in Bavaria and captures health-care data for about 91% of the female population, as fewer than 10% are privately insured. KVB receives quarterly data from office-based physicians who treat patients with statutory health insurance for reimbursement. This analysis was based on anonymised health claims and prescription data, with information on sex, age, place of residence, HPV vaccine type (Cervarix®, Gardasil®, Gardasil®9), age at vaccination, participation in J1 examination (preventive health check-up offered to adolescents aged 12–14 years and covered by statutory health insurance), cervical cancer screening, and oral contraceptive use. The variable cervical cancer screening was generated from the following German billing codes (GOP) for cervical cancer screening: 01730/01760, 01761, 01762, 01763, 01733. Prescription data contains information on prescribed medicines classified according to the Anatomical Therapeutic Chemical (ATC) system. Information on HPV vaccination status was deduced from health claims and prescription data. For data protection reasons, the date of birth was available in the month-year format. Data were solely analysed within the protected KVB server system.

### HPV vaccination

Following the recommendation of HPV vaccination in 2007, the vaccine was initially administered using a 3-dose scheme in Germany. This was updated to a 2-dose scheme for 9- to 14-year-olds in 2014, while those aged 15 years and older continued with the 3-dose scheme. This analysis defined full vaccination as two doses received at least 5 months apart for the 9 to 14 age group and three doses received from age 15, irrespective of the interval between the doses. Any deviation from this definition was considered partially vaccinated. Unvaccinated females were defined as those for whom no dose of any HPV vaccine was documented. To ensure accurate ascertainment of vaccination status, females were identified as vaccinated if an office-based physician registered in Bavaria billed an HPV vaccine according to a standard fee schedule or issued a prescription for any of the three HPV vaccines licensed in Germany (Gardasil® (Merck, Sharp & Dohme Corp.), Cervarix® (GlaxoSmithKline Biologicals), and Gardasil®9 (Merck, Sharp & Dohme Corp.)). Since late 2019, vaccines have been administered directly from the office-based physician's stock without a prescription, and therefore, the vaccine type used is not reported to the KVB. However, because Gardasil9® has been used almost exclusively in Bavaria since 2017,^25^ we assumed that individuals vaccinated from 2019 onwards received Gardasil®9. The KVB database captures vaccination data only for individuals eligible under the STIKO recommendation (9–18 years). The age at vaccination was determined by considering the first HPV vaccination date, and month and year of birth. Women who received HPV vaccination during follow-up contributed person-time to the unvaccinated group until the date of first vaccination, after which they were classified as partially vaccinated, and subsequently as fully vaccinated once the criteria for full vaccination were met. Participants who never met the criteria for full vaccination remained in the partially vaccinated group, whereas those with no recorded vaccination contributed person-time exclusively to the unvaccinated group.

### Contraceptive prescription

The use of hormonal contraceptives requires a physician's prescription in Germany, and these are documented in the prescription database of the KVB using the ATC codes AG02B and AG03A. The first prescription of contraceptives was used as a proxy for a potential start of sexual activity.

### Place of residence

The Federal Institute of Building, Urban Affairs, and Spatial Development in Germany groups district types for research purposes based on the population size and density. The four types of districts based on this classification are district-free (larger) cities (25 in number), district (smaller) cities, rural districts, and sparsely populated rural districts. In this study, the 25 district-free larger cities will be referred to as larger cities and the district (smaller) cities, rural districts, and sparsely populated rural districts will be collectively referred to as rural areas.

### Statistical analyses

The primary outcomes to be considered were confirmed precancerous lesions of the cervix uteri, including carcinoma in situ (ICD-10 N87.1, N87.2, D06) and invasive cervical cancer (ICD-10 C53). Participants with any of the primary outcomes were identified using the respective ICD code from the KVB database. To qualify as a validated invasive cervical cancer diagnosis, there must have been evidence of a pathology billing code or a confirmatory diagnostic or histological examination within the two years preceding the diagnosis, and an observation period of at least two years prior to diagnosis.

The proportion of women diagnosed with precancerous cervical lesions or invasive cervical cancer during the 17-year observation period was calculated overall and by age group (19–24, 25–29, 30–34 years). The proportions were stratified by vaccination status–fully vaccinated, partially vaccinated, and unvaccinated. For this, the number of females with a diagnosis at any time during follow-up was divided by the total number of females in the respective group. Participants were grouped according to their age at the end of the follow-up period in 2024, serving as a proxy for birth cohort. Outcomes were counted irrespective of the age at which they occurred. The 95% confidence intervals (95% CI) were computed using the z-score formula under the assumption of a normal distribution and a sufficiently large sample size. To ensure that the disease status was accurately known, we restricted this analysis population to women who had at least one contact with an office-based physician in 2024.

The cumulative incidence was computed as 1-KM, where KM represents the Kaplan–Meier estimate. Cumulative incidence curves were plotted by age at follow-up to visualise differences in the incidence of precancerous lesions and invasive cervical cancer by vaccination status, age at vaccination (≤14 years, ≥15 years), and contraceptive prescription before HPV vaccination. The curves were plotted conditionally on the assumption that all participants were event-free until age 19 years. Vaccination status was considered a fixed variable since after 18 years of age the status cannot change anymore in the KVB data.

We used Cox regression models to estimate the hazard ratios of being fully or partially vaccinated compared to being unvaccinated for both outcomes, i.e., precancerous lesions and cervical cancer. The crude model (Model 1) includes all women and estimates the hazard ratio for fully vaccinated and partially vaccinated females with the unvaccinated as the reference. The fully adjusted model (Model 2) is the main model, which adjusts model 1 for age at vaccination (≤14 years, ≥15 years) and oral contraception prescription prior to vaccination (yes/no). Additional covariables included cervical cancer screening status (ever or never screened) and place of residence (larger cities or rural areas).

For the secondary analyses, in addition to the variables in model 2, model 3 was adjusted for vaccine type. In a further secondary analysis (Model 4), analysis was restricted to the 1996–2005 birth cohort and additionally adjusts for J1 examination attendance. This model excluded older birth cohorts who were not eligible anymore for the J1 examination at the start of the analysis period. All variables in Models 1 to 4 (except for place of residence) were treated as time-dependent. Age at vaccination, contraceptive prescription prior to vaccination, and vaccine type were considered effect modifiers.

As sensitivity analysis, we then reclassified vaccination status as one dose vs two or more doses and repeated the analyses accordingly. Models 5, 6, 7, and 8 are the equivalents of models 1, 2, 3, and 4 above, respectively.

Because claims data contain mandatory fields for reimbursement, no missing data were present in the analysis. However, for 4503 individuals, the information required to classify their place of residence as a larger city or a rural area was unavailable and therefore were assigned to the “unknown” residence category. No imputation or other missing data handling methods were applied. The proportional hazards assumption was checked with Schoenfeld residuals plots. The significance level was set at 5%. All analyses were performed using the R software version 4.4.3.

### Ethical approval

In accordance with the “GPS—Good Practice in Secondary Data Analysis” (Guideline 1: Ethics),^28^ the analysis of anonymised claims data from the statutory health insurance does not require the approval of an ethics committee. The project was, however, approved by the board of directors of KVB.

### Role of funding source

This research received no specific grant from any funding agency, commercial or not-for-profit sectors.

## Results

**Fig. 1:**
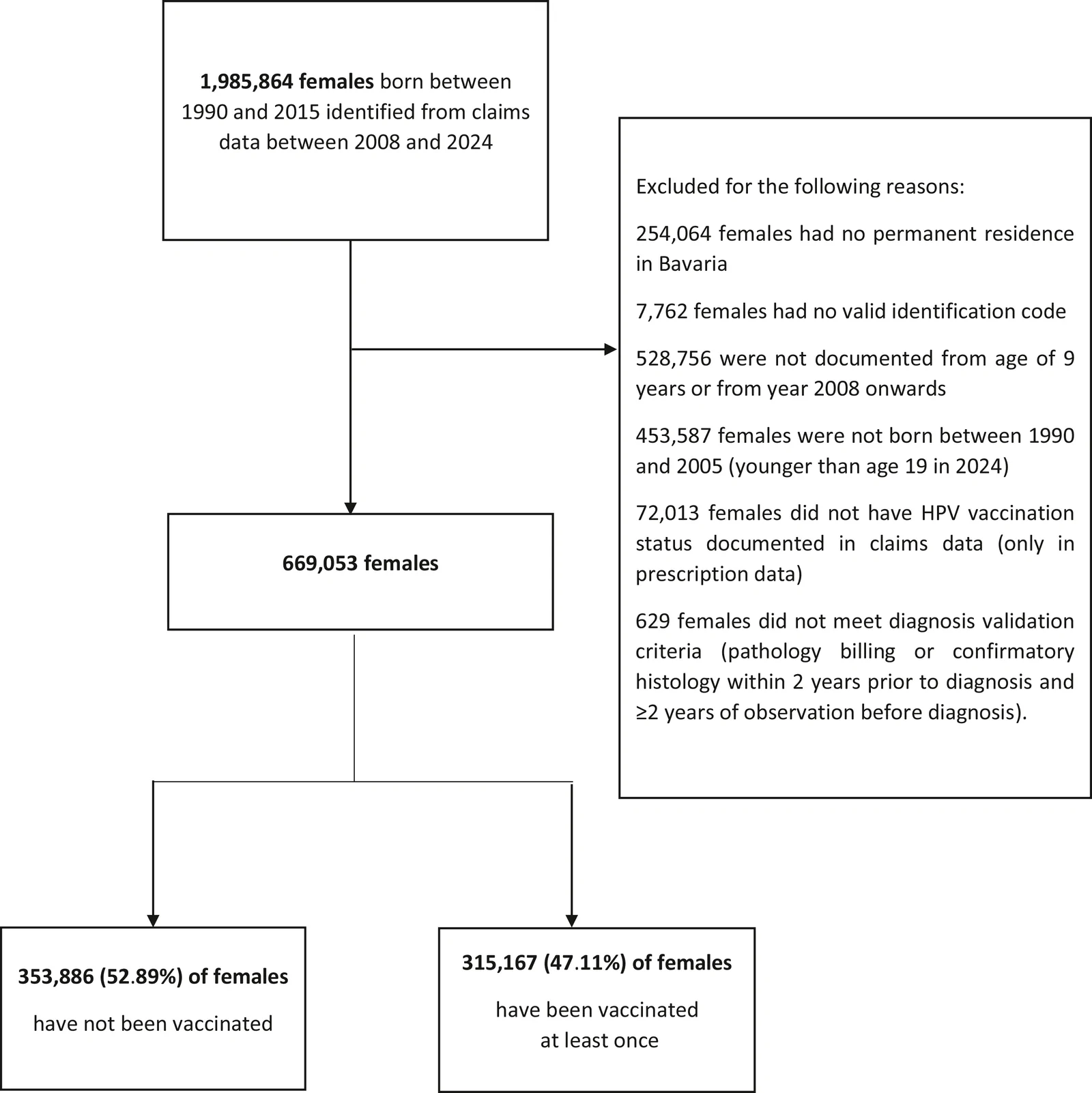
Flowchart of the analysis population using health claims and prescription data.

A total of 1,985,864 females born between 1990 and 2015 were identified from the KVB health claims data between 2008 and 2024 (Fig. 1). Of these, 669,053 met the inclusion criteria. Overall, 7.46% (49,940/669,053) women received only one dose of the HPV vaccine, 39.64% (265,227/669,053) received two or more doses, and 52.89% (353,886/669,053) were unvaccinated (Supplementary Table S1). Based on the study definitions, 33.76% (225,899/669,053) were fully vaccinated, and 13.34% (89,268/669,053) were partially vaccinated (Table 1). Therefore, 47.11% (315,167/669,053) of the study population received at least one dose of the HPV vaccine. Of those vaccinated, 49.24% (155,199/315,167) had their first vaccine at age ≥15 years, 36.85% (116,143/315,167) at ages 13–14 years, and 13.91% (43,825/315,167) by age 12 years. 84.92% (267,629/315,167) of those vaccinated had no hormonal contraceptive prescriptions prior to vaccination. Among fully vaccinated cohorts, 89.75% (202,740/225,899) had no prior prescriptions, and among partially vaccinated cohorts, 72.69% (64,889/89,268) had no prior prescriptions. 87.11% (274,530/315,167) of those vaccinated had received the Gardasil® or Gardasil®9 vaccine. Among fully vaccinated cohorts, 54.29% (122,642/225,899) belonged to the 2000–2005 birth cohort, and 10.87% (24,553/225,899) belonged to the 1990–1994 birth cohort. About 80.96% (541,642/669,053) of the study population resided in rural areas (Table 1).

**Table 1:** Characteristics of the entire female analysis population (n = 669,053) and vaccinated cohorts (fully vaccinated and partially vaccinated) only (n = 315,167).

Demographic variables	Overall	Fully vaccinated^a^	Partially vaccinated^b^	Unvaccinated^c^
	n (%^e^)	n (%^e^)	n (%^e^)	n (%^e^)
**Characteristics of the entire analysis population**				
Year of birth				
1990–1994	196,910 (29.43)	24,553 (10.87)	37,413 (41.91)	134,944 (38.13)
1995–1999	213,384 (31.89)	78,704 (34.84)	22,505 (25.21)	112,175 (31.70)
2000–2005	258,759 (38.68)	122,642 (54.29)	29,350 (32.88)	106,767 (30.17)
Place of residence				
Rural areas	541,642 (80.96)	182,761 (80.90)	71,351 (79.93)	287,530 (81.25)
Larger cities	122,908 (18.37)	43,105 (19.08)	17,856 (20.00)	61,947 (17.50)
Unknown	4503 (0.67)	33 (0.01)	61 (0.07)	4409 (1.25)
Cervical Cancer Screening				
No	194,913 (29.13)	70,885 (31.38)	21,755 (24.37)	102,273 (28.90)
Yes	474,140 (70.87)	155,014 (68.62)	67,513 (75.63)	251,613 (71.10)
J1 examination^f^				
No	267,413 (39.97)	93,064 (41.20)	28,663 (32.11)	145,686 (41.17)
Yes	165,117 (24.68)	96,237 (42.60)	17,420 (19.51)	51,460 (14.54)
Not applicable	236,523 (35.35)	36,598 (16.20)	43,185 (48.38)	156,740 (44.29)
Total	669,053 (100.00)	225,899 (33.76)	89,268 (13.34)	353,886 (52.89)
**Characteristics of vaccinated cohorts only**^d^				
Age at vaccination				
Up to 12 years	43,825 (13.91)	38,188 (16.90)	5637 (6.31)	
13–14 years	116,143 (36.85)	94,387 (41.78)	21,756 (24.37)	
15 years and above	155,199 (49.24)	93,324 (41.31)	61,875 (69.31)	
Contraceptive prescription before vaccination				
No	267,629 (84.92)	202,740 (89.75)	64,889 (72.69)	
Yes	47,538 (15.08)	23,159 (10.25)	24,379 (27.31)	
Vaccine type				
Cervarix®	22,647 (7.19)	17,300 (7.66)	5347 (5.99)	
Gardasil®/Gardasil®9	274,530 (87.11)	196,766 (87.10)	77,764 (87.11)	
Unknown	17,990 (5.71)	11,833 (5.24)	6157 (6.90)	
Total	315,167 (100.00)	225,899 (100.00)	89,268 (100.00)	

n: number of females.

a Fully vaccinated was defined as two doses of vaccine received at least 5 months apart for the 9–14 age group and three doses received from age 15 years, irrespective of the interval between the doses.

b Any deviation from the fully vaccinated definition was considered as partially vaccinated.

c Unvaccinated females were those who had never received a dose of any HPV vaccine.

d At least one dose of any HPV vaccine.

e Percentages may not total 100 because of rounding.

f J1 examination status was only assessed in the 1996–2005 birth cohort (n = 432,530).

### Precancerous lesions

8965 diagnoses of precancerous lesions (ICD-10 N87.1, N87.2, D06) in women aged 19–34 years were documented during the 17-year study period (n = 541,270), with an overall proportion of women with a diagnosis of 1.66% (95% CI: 1.62%, 1.69%) at the end of the follow-up period (Table 2). The proportion of women with a diagnosis at the end of follow-up in the fully vaccinated, partially vaccinated, and unvaccinated cohorts were 0.86% (95% CI: 0.82%, 0.90%), 1.55% (95% CI: 1.46%, 1.63%), and 2.27% (95% CI: 2.21%, 2.32%), respectively. Overall, the proportion of women with a diagnosis increased with age.

**Table 2:** Proportion of women diagnosed with precancerous lesions and cervical cancer during the 17-year observation period (2008–2024), by age at end of follow-up (2024), stratified by vaccination status (fully vaccinated, partially vaccinated, unvaccinated) (n = 541,270^d^).

Primary outcomes		Proportion % (95% confidence interval)			
		Overall	Fully vaccinated^a^	Partially vaccinated^b^	Unvaccinated^c^
n		541,270	196,098	74,320	270,852
**Precancerous lesions**					
Age^e^	n at-risk (no. of cases)				
19–24	218,141 (769)	0.35 (0.33–0.38)	0.28 (0.25–0.31)	0.35 (0.28–0.43)	0.45 (0.41–0.50)
25–29	171,060 (2908)	1.70 (1.64–1.76)	1.42 (1.33–1.51)	1.65 (1.47–1.84)	1.92 (1.83–2.01)
30–34	152,069 (5288)	3.48 (3.39–3.57)	2.19 (1.99–2.40)	2.52 (2.34–2.70)	4.00 (3.88–4.12)
Overall	541,270 (8965)	1.66 (1.62–1.69)	0.86 (0.82–0.90)	1.55 (1.46–1.63)	2.27 (2.21–2.32)
**Cervical cancer**					
Age^e^	n at-risk (no. of cases)				
19–24	218,141 (33)	0.02 (0.01–0.02)	0.01 (0.01–0.02)	0.03 (0.01–0.05)	0.01 (0.01–0.02)
25–29	171,060 (172)	0.10 (0.09–0.12)	0.08 (0.06–0.10)	0.07 (0.03–0.11)	0.12 (0.10–0.15)
30–34	152,069 (464)	0.31 (0.28–0.33)	0.16 (0.11–0.22)	0.20 (0.15–0.26)	0.36 (0.33–0.40)
Overall	541,270 (669)	0.12 (0.11–0.13)	0.05 (0.04–0.06)	0.11 (0.09–0.13)	0.18 (0.16–0.20)

n: number of females.

a Fully vaccinated was defined as two doses of vaccine received at least 5 months apart for the 9–14 group and three doses received from age 15 years, irrespective of the interval between the doses.

b Any deviation from the fully vaccinated definition was considered as partially vaccinated.

c Unvaccinated persons were those who had never received a dose of any HPV vaccine.

d For the calculation of the proportions, the population was limited to those who contacted a physician in 2024.

e Participants were grouped according to their age at the end of follow-up in 2024; outcomes were counted irrespective of the age at diagnosis.

**Fig. 2:**
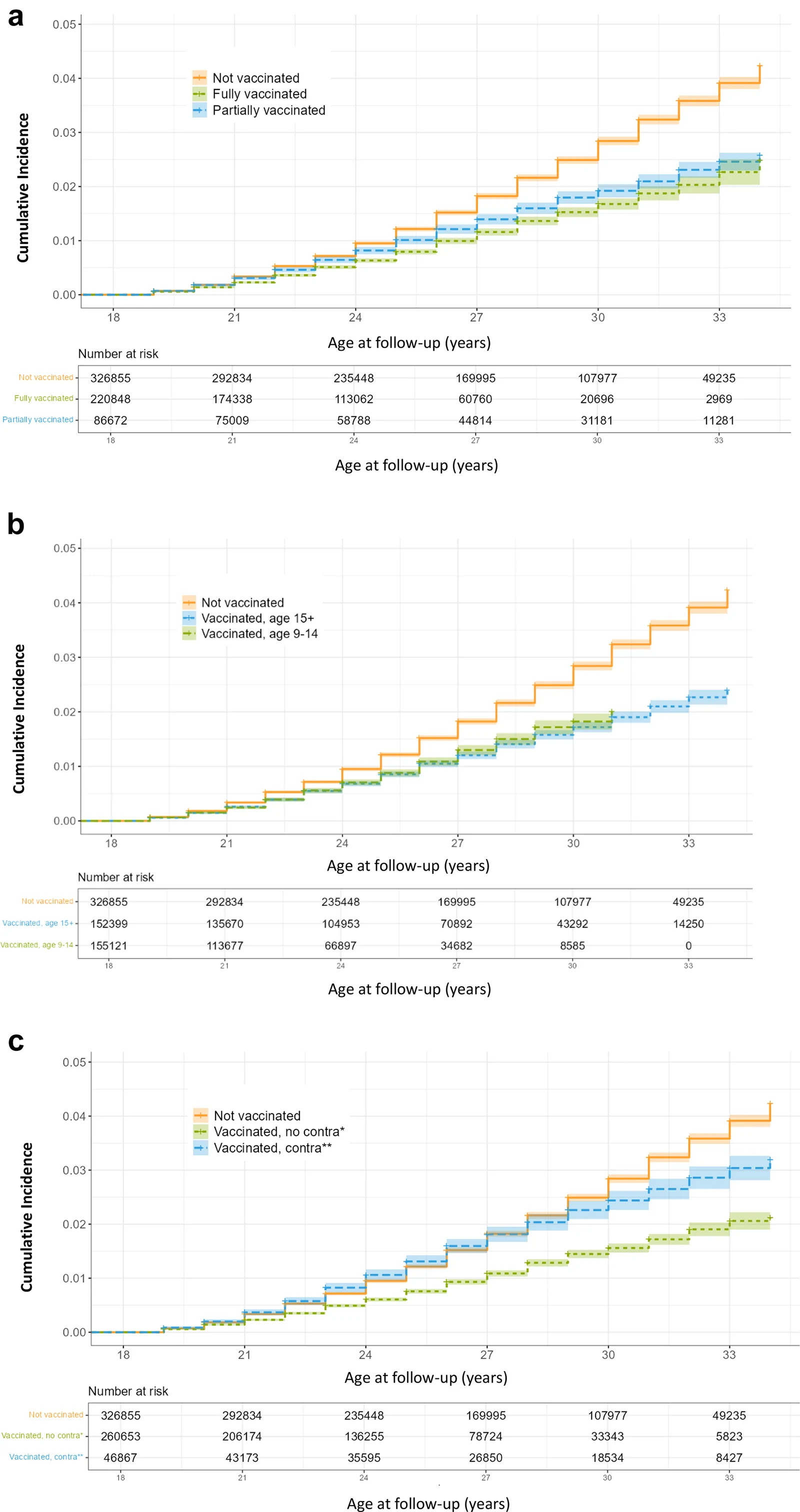
Cumulative incidence of precancerous lesions (ICD-10 N87.1, N87.2, D06) by age at follow-up stratified by a) Vaccination status, b) Age at vaccination, and c) Contraception prescription prior to vaccination in females from 9 to 34 years in Bavaria (n = 669,053).Definition: Fully vaccinated was defined as receiving two doses of vaccine at least 5 months apart for the 9–14 age group and three doses for individuals aged 15 years or older, irrespective of the interval between doses. Any deviation from this definition was considered partially vaccinated. Unvaccinated females were those who had never received a dose of any HPV vaccine. ∗ no contra: no contraceptives prescription before vaccination; ∗∗ contra: contraceptives prescription before vaccination.

Fig. 2 shows the cumulative incidence curves for precancerous lesions by age at follow-up. The probability of diagnosis of precancerous lesions by age 34 was 0.025 (95% CI: 0.020, 0.029) in the fully vaccinated group, 0.025 (95% CI: 0.024, 0.027) in the partially vaccinated group, and 0.041 (95% CI: 0.040, 0.043) in the unvaccinated group (Fig. 2A, Supplementary Table S6). Cohorts vaccinated before 15 years had a cumulative incidence of 0.020 (95% CI: 0.017, 0.023) by age 31 years and those vaccinated from 15 years had a cumulative incidence of 0.024 (95% CI: 0.022, 0.025) by 34 years (Fig. 2B, Supplementary Table S7). Among vaccinated cohorts with hormonal contraception prescription prior to vaccination, the probability of diagnosis was 0.031 (95% CI: 0.029, 0.034) by age 34 (Fig. 2C, Supplementary Table S7).

The crude hazard ratios (n = 669,053) were 0.63 (95% CI: 0.60, 0.67) for fully vaccinated and 0.70 (95% CI: 0.66, 0.75) for partially vaccinated females compared to unvaccinated (Table 3). After full adjustment, the hazard ratio was 0.48 (95% CI: 0.45, 0.52) among fully vaccinated women and 0.53 (95% CI: 0.49, 0.57) among partially vaccinated females compared to unvaccinated (Table 3). The risk was higher among females living in larger cities (aHR: 1.24; 95% CI: 1.18, 1.31), among those who received hormonal contraceptives before vaccination (aHR: 1.58; 95% CI: 1.45, 1.73), those vaccinated before 15 years (aHR: 1.34; 95% CI: 1.23, 1.46) and among those who had ever been screened (aHR: 6.97; 95% CI: 6.27, 7.75).

**Table 3:** Crude and adjusted Cox proportional hazard models on the association of HPV vaccination status (fully vaccinated, partially vaccinated, unvaccinated) and other factors on the risk of diagnosis of precancerous lesions and invasive cervical cancer in females (n = 669,053).

Covariable	Precancerous lesions Hazard ratio (95% confidence interval)	Cervical cancer Hazard ratio (95% confidence interval)
**Crude model (Model 1)**^a^		
HPV vaccination status		
Partially vaccinated^d^ (Ref. Unvaccinated^e^)	0.70 (0.66–0.75)	0.60 (0.48–0.76)
Fully vaccinated^c^ (Ref. Unvaccinated)	0.63 (0.60–0.67)	0.46 (0.36–0.57)
**Fully adjusted model (Model 2)**^b^		
HPV vaccination status		
Partially vaccinated (Ref. Unvaccinated)	0.53 (0.49–0.57)	0.40 (0.29–0.55)
Fully vaccinated (Ref. Unvaccinated)	0.48 (0.45–0.52)	0.35 (0.26–0.47)
Cervical cancer screening		
Yes (Ref. No)	6.97 (6.27–7.75)	4.86 (3.29–7.18)
Age at vaccination		
9–14 years (Ref. ≥15 years)	1.34 (1.23–1.46)	1.16 (0.80–1.66)
Contraception before HPV vaccination		
Yes (Ref. No)	1.58 (1.45–1.73)	2.13 (1.51–3.00)
Place of residence^f^		
Larger cities (Ref. Rural areas)	1.24 (1.18–1.31)	1.28 (1.06–1.53)

Ref.: Reference.

a Model 1 is the crude model, includes all females, and evaluates the association between HPV vaccination status and the risk of diagnosis.

b Model 2 is the fully adjusted model and includes all females, assesses the association between HPV vaccination status and the risk of diagnosis, and adjusts for cervical cancer screening participation, age at vaccination, place of residence, and contraceptive prescription prior to vaccination.

c Fully vaccinated was defined as two doses of vaccine received at least 5 months apart for the 9–14 group and three doses received from age 15 years, irrespective of the interval between the doses.

d Any deviation from the fully vaccinated definition was considered as partially vaccinated.

e Unvaccinated persons were those who had never received a dose of any HPV vaccine.

f Place of residence: larger cities (district-free larger cities) vs rural areas (district cities, rural districts, and sparsely populated rural districts) in Bavaria.

### Cervical cancer

669 females were diagnosed with cervical cancer (ICD-10 C53) during the 17-year study period (n = 541,270), resulting in an overall proportion of women with a diagnosis of 0.12% (95% CI: 0.11%, 0.13%) at the end of the follow-up period (Table 2). The proportion of women with a diagnosis at the end of the follow-up was 0.05% (95% CI: 0.04%, 0.06%) in the fully vaccinated cohort, 0.11% (95% CI: 0.09%, 0.13%) in the partially vaccinated cohort, and 0.18% (95% CI: 0.16%, 0.20%) in the unvaccinated cohort. Overall, the proportion of women with a diagnosis increased with age.

**Fig. 3:**
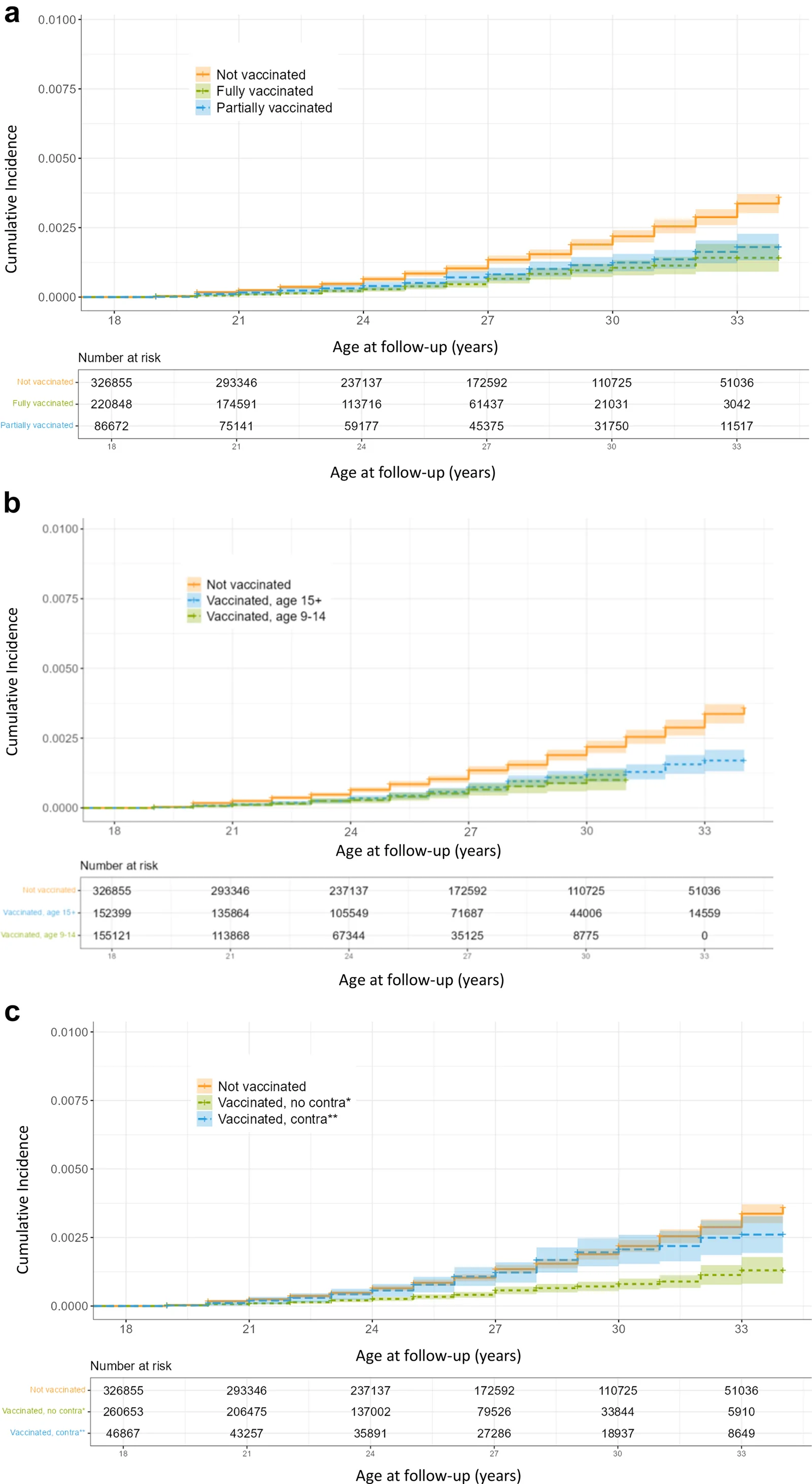
Cumulative incidence of cervical cancer (ICD-10 C53) by age at follow-up stratified by a) Vaccination status, b) Age at vaccination, and c) Contraception prescription prior to vaccination in females from 9 to 34 years in Bavaria (n = 669,053). Definition: Fully vaccinated was defined as receiving two doses of vaccine at least 5 months apart for the 9–14 age group and three doses for individuals aged 15 years or older, irrespective of the interval between doses. Any deviation from this definition was considered partially vaccinated. Unvaccinated females were those who had never received a dose of any HPV vaccine. ∗ no contra: no contraceptives prescription before vaccination; ∗∗ contra: contraceptives prescription before vaccination.

Fig. 3 shows the cumulative incidence curves for cervical cancer by age at follow-up. By age 34, the probability of diagnosis of cervical cancer was 0.001 (95% CI: 0.001, 0.002) in the fully vaccinated cohort, 0.002 (95% CI: 0.001, 0.002) in the partially vaccinated cohort, and 0.004 (95% CI: 0.003, 0.004) in the unvaccinated cohort (Fig. 3A, Supplementary Table S6). Cohorts vaccinated before 15 years of age had a cumulative incidence of 0.001 (95% CI: 0.001, 0.001) by age 31 years, and those vaccinated from 15 years had a cumulative incidence of 0.002 (95% CI: 0.001, 0.002) by age 34 years (Fig. 3B, Supplementary Table S7). The probability of diagnosis with cervical cancer was higher in vaccinated cohorts with hormonal contraceptive prescriptions issued prior to vaccination and reached 0.003 (95% CI: 0.002, 0.003) by age 34 (Fig. 3C, Supplementary Table S7).

The crude hazard ratios (n = 669,053) were 0.46 (95% CI: 0.36, 0.57) and 0.60 (95% CI: 0.48, 0.76) for fully and partially vaccinated cohorts, respectively, compared to the unvaccinated (Table 3). The adjusted hazard ratios for cervical cancer in fully vaccinated and partially vaccinated females were 0.35 (95% CI: 0.26, 0.47) and 0.40 (95% CI: 0.29, 0.55), respectively. The hazard ratio for cervical cancer was 1.28 (95% CI: 1.06, 1.53) for those living in larger cities, 2.13 (95% CI: 1.51, 3.00) for those who had a contraceptive prescription before vaccination, and 4.86 (95% CI: 3.29, 7.18) for those who had ever been screened. Age at vaccination had no significant effect on the risk of diagnosis (aHR: 1.16; 95% CI: 0.80, 1.66).

### Secondary analysis considering vaccine type and J1 examination attendance

The use of Cervarix® was associated with a lower risk of precancerous lesions compared with Gardasil® or Gardasil®9 (aHR: 0.86, (95% CI 0.75, 0.98)), whereas no statistically significant difference was observed for invasive cervical cancer (aHR: 0.65, (95% CI: 0.35, 1.24)) (Supplementary Table S5). Attendance at the J1 examination was not significantly associated with risk of precancerous lesions (aHR: 0.94, (95% CI: 0.87, 1.01)) or invasive cervical cancer (aHR: 1.14, (95% CI: 0.84, 1.56)) among cohorts born between 1996 and 2005 (n = 432,530).

### Analysis one dose vs two or more doses of HPV vaccine

For precancerous lesions, the proportion of women with a diagnosis over the 17-year period was 1.67% (95% CI: 1.54%, 1.79%) and 0.94% (95% CI: 0.90%, 0.97%) for those who received one dose and two or more doses, respectively (Supplementary Table S2). Cohorts who received one dose of the vaccine had a cumulative incidence of 0.025 (95% CI: 0.023, 0.027) and those who received two or more doses had a cumulative incidence of 0.024 (95% CI: 0.022, 0.027) (Supplementary Figure S1A, Supplementary Table S6). The crude hazard ratios were 0.79 (95% CI: 0.75, 0.84) and 0.70 (95% CI: 0.66, 0.73) for one dose and two or more doses, respectively, and adjusted hazard ratios were 0.68 (95% CI: 0.64, 0.73) and 0.60 (95% CI: 0.57, 0.64) (Supplementary Table S3).

For cervical cancer, the proportion of women with a diagnosis over the 17-year period was 0.12% (95% CI: 0.09%, 0.15%) and 0.06% (95% CI: 0.05%, 0.07%) for those who received one dose and two or more doses, respectively (Supplementary Table S2). Cohorts who received one dose of the vaccine had a cumulative incidence of 0.002 (95% CI: 0.001, 0.002) and those who received two or more doses had a cumulative incidence of 0.002 (95% CI: 0.001, 0.002) (Supplementary Figure S1B, Supplementary Table S6). The crude hazard ratios were 0.66 (95% CI: 0.52, 0.83) and 0.53 (95% CI: 0.44, 0.64) for one dose and two or more doses groups, respectively, and adjusted hazard ratios were 0.50 (95% CI: 0.38, 0.66) and 0.45 (95% CI: 0.34, 0.57) (Supplementary Table S3).

In secondary analyses, neither vaccine type nor J1 attendance was significantly associated with precancerous lesions or cervical cancer (Supplementary Table S4).

## Discussion

This retrospective cohort analysis included 669,053 Bavarian females aged 19–34 years. Of the 541,270 women who had at least one contact with an office-based physician in 2024, the overall proportion of women with a diagnosis at the end of the follow-up period was 1.66% for precancerous lesions and 0.12% for invasive cervical cancer. This proportion increased with age and was consistently higher among unvaccinated individuals than among their vaccinated counterparts. By age 34 years, the cumulative incidence of both precancerous cervical lesions and invasive cervical cancer was lower in vaccinated cohorts than in unvaccinated cohorts. Cox regression analyses showed a 52% and 65% lower risk of being diagnosed with precancerous lesions and invasive cervical cancer, respectively, in those who were fully vaccinated compared to unvaccinated. Partially vaccinated cohorts had a 47% and 60% reduced risk of being diagnosed with precancerous lesions and cervical cancer, respectively. For precancerous lesions, vaccination before 15 years of age was associated with a higher risk of diagnosis of precancerous lesions, while for cervical cancer, there was no statistically significant effect of age at vaccination on diagnosis risk. For both outcomes, hormonal contraceptive prescriptions before vaccination, residing in larger cities, and having ever been screened for cervical cancer were associated with a statistically significantly increased risk of diagnosis.

The results from our analysis support previous real-world effectiveness studies,^21^, ^22^, ^23^, ^24^ which have shown that HPV vaccination was effective in reducing cervical cancer incidence. Cohort studies in Sweden,^21^ Denmark,^22^ and Scotland,^23^ report incidence risk reductions of approximately 80% or more in cohorts vaccinated before the age of 17, with the Scottish study reporting no cases of cervical cancer among those vaccinated before 14 years.^23^ In contrast to these cohort studies, the reduction observed in our study is markedly lower, at 65%. With comparatively lower vaccination coverage in our study population (47.11%) and likely weaker herd effects than in Sweden, Denmark, or Scotland, which have achieved vaccination rates of 80% or more,^14^ a larger difference in risk reduction might have been expected. Reasons for this finding might lie in the differences in age at vaccination and sexual behaviour between the populations.

Half of our vaccinated population received their first vaccine at age 15 years or older, while only 13.91% were vaccinated before age 13, and therefore, a substantial proportion may have received the vaccine after their sexual debut. In contrast, in Sweden and Scotland, which have school-based vaccination programs, 80–90% of girls are vaccinated before age 14.^12^^,^^13^ A German cross-sectional survey conducted between 2014 and 2017 involving almost 3000 females aged 14–31 years found that 36% of the respondents had initiated sexual activity by age 15.^29^ In Sweden, on the other hand, only about 18% of cohorts born between 1989 and 1994 had their first sexual activity by age 15. Since an increase in HPV infections is observed shortly after sexual debut, HPV vaccination prior to this provides the greatest protection against HPV-related cancer.^30^ Effectiveness, however, decreases with vaccination after the sexual debut.^30^ Our results align with this evidence, showing reduced HPV vaccine effectiveness in individuals with prior hormonal contraceptive prescriptions, used here as a proxy for sexual debut. Consequently, the WHO^5^ and the German STIKO^8^ recommend vaccination between ages 9 and 14, while the American ACIP^6^ recommends vaccination at 11 or 12 years, when sexual activity has likely not started yet. However, we did not observe a significant association between age at vaccination and risk of invasive cervical cancer after adjusting for pre-vaccination contraception prescription, which may better capture the timing of sexual debut. The inconsistent association between age at vaccination and the risk of cervical precancerous lesions or invasive cervical cancer should, however, be interpreted with caution, given the possibility of residual confounding, the correlation of this variable with oral contraceptive use, and the limited data on sexual behaviour.

Precancerous cervical lesions and early stages of cervical cancer are asymptomatic, and their detection depends on screening uptake and screening quality. In our population, cervical cancer screening participation was similar between vaccinated and unvaccinated groups, although vaccinated women have been shown to participate more frequently in screening than unvaccinated women.^31^ The overall cervical cancer screening participation rate was 70.87% in our study population, with only minimal differences between vaccinated and unvaccinated groups. By adjusting for screening participation, we account for these minor differences. The higher risk of precancerous lesions observed with earlier vaccination in our study is likely due to differences in screening timing rather than true differences in disease risk, as females vaccinated at younger ages may undergo screening earlier or more frequently, leading to earlier detection of asymptomatic lesions and seemingly higher risk.

In our study, fully vaccinated cohorts had a 52% and a 65% lower risk of developing precancerous lesions and cervical cancer, respectively, compared to the unvaccinated. The observed greater protection of the HPV vaccine for cervical cancer compared to precancerous lesions was also reported for Denmark.^22^ This has been attributed to the differences in the causal HPV types for the two diagnoses. In Europe, approximately 80% of cervical cancers are attributable to HPV types 16 and 18.^32^ These types account for only about 55% of precancerous lesions, with other oncogenic types, particularly HPV 31 and 33, contributing substantially.^33^ In Germany, the quadrivalent Gardasil®, which does not cover HPV types 31 and 33, was the primary vaccine used for vaccination until the introduction of the nonavalent Gardasil®9 in 2016, which then became the primary vaccine administered in 2017.^25^ The bivalent Cervarix® has always been the least administered vaccine, and its use has decreased drastically over the years in Germany.^25^ Since the nonavalent vaccine is currently used, future studies may demonstrate higher protection against precancerous lesions.

Our analysis consistently showed lower risks of precancerous lesions (52% vs 47%) and cervical cancer (65% vs 60%) in fully vaccinated cohorts compared to their partially vaccinated counterparts, as well as for two and more doses of the vaccine compared to one dose only (precancerous lesions: 40% vs 32%, cervical cancer: 55% vs 50%). However, the confidence intervals overlap. This is consistent with the IARC single-dose HPV vaccine efficacy trial, which included unmarried women aged 10–18 years with a 12-year follow-up,^34^ and reported slightly higher vaccine efficacy with increasing numbers of doses, although the differences between one-, two-, and three-dose regimens of the quadrivalent HPV vaccine were small and not statistically significant. The KENSHE randomised controlled trial among Kenyan women aged 15–20 years showed high efficacy and durable protection after a single dose of the bivalent or nonavalent HPV vaccine.^35^ Similar results were reported from the Costa Rica trial using the bivalent vaccine, which enrolled women aged 18–25 years and had a median follow-up of 11 years, although antibody titres were lower in the one-dose group compared to the two- and three-dose groups.^36^ The KENSHE and Costa Rica trials focused on vaccine-type–specific infection and immunological responses, whereas our study assessed clinically diagnosed precancerous lesions and invasive cervical cancer. A Scottish cohort study,^23^ also reported a higher cervical cancer incidence among partially vaccinated girls compared to the fully vaccinated when vaccination occurred at age 14 years or older after a 12-year follow-up period. There was, however, no difference in incidence between partially and fully vaccinated cohorts vaccinated at ages 12–13 in Scotland.^23^

J1 examination is a preventive health check-up offered to adolescents aged 12–14 years, usually accompanied by their parents, and is covered by statutory health insurance in Germany. It includes a physical examination and an assessment of vaccination status. A 2025 review of KVB data showed overall low participation in J1 but found that attendance was associated with a higher likelihood of HPV vaccination and completion of the vaccine series.^37^ In our study, J1 participation was similarly associated with vaccination uptake but was not significantly associated with the risk of precancerous lesions or cervical cancer.

### Strengths and limitations

This study is the first to investigate the direct effects of HPV vaccination on cervical cancer incidence in Germany. Previous studies were ecological^27^ or focused on genital warts and precancerous lesions.^25^^,^^26^ A key strength of our study is the use of health claims data that covers about 91% of the female population in Bavaria, a German state with 13 million inhabitants. In the absence of comprehensive population-based vaccination and screening registries, claims data provide access to a large and representative dataset for epidemiological analyses.

However, the KVB data, like any other claims data, are primarily collected for reimbursement purposes and therefore only include statutorily insured individuals who visit office-based physician practices. This limitation is likely less pronounced in Germany, where approximately 90% of insured women aged 18 years and above have at least one practice contact per year,^38^ indicating broad population coverage. Nevertheless, the data might contain incorrect or tentative diagnoses, potentially leading to an overestimation of the proportion of women diagnosed. The KVB database does not cover privately insured individuals who might have a higher socioeconomic status. We, however, did not find any evidence in the literature examining differences in vaccination rates between privately insured and statutorily insured females.

While we use hormonal contraceptive prescriptions as a proxy for sexual debut, it is important to note that hormonal contraceptives can also be used for other medical conditions, such as the treatment of menstrual disorders. Additionally, the use of oral contraceptives is considered a risk for the development of cervical cancer. The use of other contraceptives such as condoms does not require a prescription, and therefore, individuals who use them could not be captured in our data. Moreover, while oral contraceptives were historically the most used method, condoms have become increasingly popular in Germany, especially among young people.^29^

Finally, residual confounding cannot be excluded, as important factors such as socioeconomic status and sexual behaviour are not available in this database.

### Conclusion

We showed for the first time that HPV vaccination was associated with substantial reductions in the risk of precancerous lesions and cervical cancer in a country with relatively lower HPV vaccination coverage. However, compared with countries with high vaccination coverage and stronger herd immunity, the risk reduction is lower in our analyses. Understanding the reasons for this and implementing effective strategies to improve vaccine impact will be essential to reduce the burden of HPV-related disease and achieve the WHO elimination targets. Continued monitoring of HPV vaccine effectiveness is crucial to determine whether one dose or complete schedules provide equivalent high and long-term protection, informing future vaccination strategies.

## Contributors

SJK conceptualised the study with MT. The research methodology was designed by SJK, GS, GBA and SF. SF conducted the statistical analyses with support of GS. GBA summarised the results in consultation with SF and GS. GBA, GS, SJK, MT and SF interpreted the analysis. SJK provided the resources and supervised the project. GBA wrote the original draft, and SJK, MT, SF, GS, MR, VO, and MK critically reviewed and edited it. All authors approved the final version of the manuscript. SF and MT had full access to the raw data in the study and verified the data. SJK had final responsibility for the decision to submit for publication.

## Data sharing statement

The data on which these analyses were conducted are available from the KVB (Email: info@kvb.de), but restrictions apply to the availability of these data.

## Declaration of interests

Prof. Kiechle reports research funding to the institution, consulting fees, honoraria for lectures and educational activities, travel support, and stock ownership from or in entities as detailed in the ICMJE disclosure form. These relationships are outside the submitted work. All other authors declare no competing interests.
