# Supplementary material for: The effect of HPV vaccination on precancerous lesions and invasive cervical cancer in a low-vaccination-coverage setting in Germany: a retrospective cohort analysis
Source: Lancet Reg Health Eur. 2026 Aug 26;69:101830. doi: 10.1016/j.lanepe.2026.101830 (PMC13544387; doi:10.1016/j.lanepe.2026.101830)
Supplement: Supplementary file 1 [file mmc1.docx]

**Supplementary Material**

**The effect of HPV vaccination on precancerous lesions and invasive cervical cancer in a low-vaccination-coverage setting in Germany: A retrospective cohort analysis**

Gifty Baffour Awuah, MBChB^1,3^, Sandra Fett, Dipl-Kffr^2^, Gunther Schauberger, PhD^1^, Vanesa Osmani, PhD^1^, Martin Tauscher, PhD^2^, Marianne Röbl-Mathieu, MD^4^, Prof. Marion Kiechle MD^3^, Prof. Stefanie J. Klug, PhD^1^

^1^Technical University of Munich, TUM School of Medicine and Health, Chair of Epidemiology, Munich, Germany

^2^The Bavarian Association of Statutory Health Insurance Physicians (KVB), Munich, Germany

^3^Technical University of Munich, TUM School of Medicine and Health, Department of Obstetrics and Gynaecology, Munich, Germany

^4^Gynaecologist’s Office, Adams-Lehmann-Str. 36, Munich, Germany

Correspondence address

Prof. Stefanie J. Klug, PhD, MPH

Chair of Epidemiology

TUM School of Medicine and Health

Technical University of Munich (TUM)

Am Olympiacampus 11, 80809 Munich, Germany

sekretariat.klug.epidemiologie@mh.tum.de

Tel: +49 (89) 289 24951

**Table of Contents**

[**Supplementary Table S1: Characteristics of the entire female analysis population (n=669,053) and vaccinated cohorts (two or more doses and one dose) only (n=315,167)**	3](#_Toc236345220)

[**Supplementary Table S2: Proportion of women diagnosed with precancerous lesions and cervical cancer during the 17-year observation period (2008-2024), by age at end of follow-up (2024), stratified by vaccination status (two or more doses, one dose, unvaccinated) (n=541,270#)**	5](#_Toc236345221)

[**Supplementary Table S3: Crude and adjusted Cox proportional hazards models on the association of HPV vaccination status (two or more doses, one dose, unvaccinated) and other factors on the risk of diagnosis of precancerous lesions and invasive cervical cancer in females (n=669,053)**	6](#_Toc236345222)

[**Supplementary Table S4: Adjusted Cox proportional hazards models on the association of HPV vaccination status (two or more doses, one dose, unvaccinated) and other factors on the risk of diagnosis of precancerous lesions and invasive cervical cancer in females (Model 7: n=669,053, Model 8: n=432,530).**	7](#_Toc236345223)

[**Supplementary Table S5: Adjusted Cox proportional hazards models on the association of HPV vaccination status (fully vaccinated, partially vaccinated, unvaccinated) and other factors on the risk of diagnosis of precancerous lesions and invasive cervical cancer in females (Model 3: n= 669,053, Model 4: n=432,530)**	8](#_Toc236345224)

[**Supplementary Table S6: Cumulative incidence estimates of precancerous lesions and invasive cervical cancer, stratified by vaccination status**	9](#_Toc236345225)

[**Supplementary Table S7: Cumulative incidence estimates of precancerous lesions and invasive cervical cancer, stratified by age at vaccination and contraceptive prescription status, in vaccinated cohorts only**	11](#_Toc236345226)

[**Supplementary Figure S1: Cumulative incidence of A) Precancerous lesions (ICD-10 N87·1, N87·2, D06) and B) Cervical cancer (ICD-10 C53) by age at follow-up stratified by vaccination status in females from 9-34 years in Bavaria (n=669,053).**	13](#_Toc236345227)

# **Supplementary Table S1: Characteristics of the entire female analysis population (n=669,053) and vaccinated cohorts (two or more doses and one dose) only (n=315,167)**

|  | **Overall** | **Two or more doses*** | **One Dose**** | **Unvaccinated***** |
| --- | --- | --- | --- | --- |
| **Demographic variables** | n (%^#^) | n (%^#^) | n (%^#^) | n (%^#^) |
| **Characteristics of the entire analysis population** | | | | |
| **Year of birth** |  |  |  |  |
| 1990 to 1994 | 196,910 (29·43) | 35,696 (13·46) | 26,270 (52·60) | 134,944 (38·13) |
| 1995 to 1999 | 213,384 (31·89) | 91,449 (34·48) | 9,760 (19·54) | 112,175 (31·70) |
| 2000 to 2005 | 258,759 (38·68) | 138,082 (52·06) | 13,910 (27·85) | 106,767 (30·17) |
| **Place of residence** |  |  |  |  |
| Rural areas | 541,642 (80·96) | 214,296 (80·80) | 39,816 (79·73) | 287,530 (81·25) |
| Larger cities | 122,908 (18·37) | 50,887 (19·19) | 10,074 (20·17) | 61,947 (17·50) |
| Unknown | 4,503 (0·67) | 44 (0·02) | 50 (0·10) | 4,409 (1·25) |
| **Cervical Cancer Screening** | |  |  |  |
| No | 194,913 (29·13) | 80,896 (30·50) | 11,744 (23·52) | 102,273 (28·90) |
| Yes | 474,140 (70·87) | 184,331 (69·50) | 38,196 (76·48) | 251,613 (71·10) |
| **J1 examination†** |  |  |  |  |
| No | 267,413 (39·97) | 108,888 (41·05) | 12,839 (25·71) | 145,686 (41·17) |
| Yes | 165,117 (24·68) | 106,158 (40·03) | 7,499 (15·02) | 51,460 (14·54) |
| Not applicable | 236,523 (35·35) | 50,181 (18·92) | 29,602 (59·28) | 156,740 (44·29) |
| **Total** | 669,053 (100·00) | 265,227 (39·64) | 49,940 (7·46) | 353,886 (52·89) |
| **Characteristics of Vaccinated Cohorts Only****** | | | | |
| **Age at vaccination** |  |  |  |  |
| Up to 12 years | 43,825 (13·91) | 40,506 (15·27) | 3,319 (6·65) |  |
| 13 to 14 years | 116,143 (36·85) | 102,433 (38·62) | 13,710 (27·45) |  |
| 15 years and above | 155,199 (49·24) | 122,288 (46·11) | 32,911 (65·90) |  |
| **Contraceptive prescription before vaccination** | | |  |  |
| No | 267,629 (84·92) | 232,613 (87·70) | 35,016 (70·12) |  |
| Yes | 47,538 (15·08) | 32,614 (12·30) | 14,924 (29·88) |  |
| **Vaccine type** |  |  |  |  |
| Cervarix® | 22,647 (7·19) | 19,863 (7·49) | 2,784 (5·57) |  |
| Gardasil®/Gardasil®9 | 274,530 (87·11) | 229,794 (86·64) | 44,736 (89·58) |  |
| Unknown | 17,990 (5·71) | 15,570 (5·87) | 2,420 (4·85) |  |
| **Total** | 315,167 (100·00) | 265,227 (100·00) | 49,940 (100·00) |  |

* Two or more doses was defined as at least two doses of HPV vaccine received, irrespective of the interval between the doses.

** One dose was defined as receiving one dose of the HPV vaccine.

*** Unvaccinated females were those who had never received a dose of any HPV vaccine.

**** At least one dose of any HPV vaccine

n: number of females

^#^ Percentages may not total 100 because of rounding

† J1 examination status was only assessed in the 1996-2005 birth cohort (n=432,530)

# **Supplementary Table S2: Proportion of women diagnosed with precancerous lesions and cervical cancer during the 17-year observation period (2008-2024), by age at end of follow-up (2024), stratified by vaccination status (two or more doses, one dose, unvaccinated) (n=541,270#)**

|  | | Proportion % (95% Confidence Interval) | | | | |
| --- | --- | --- | --- | --- | --- | --- |
| Primary outcomes | | **Overall** | **Two or more doses*** | **One dose**** | | **Unvaccinated***** |
| n | | 541,270 | 229,322 | 41,096 | 270,852 | |
| **Precancerous lesions** | |  |  |  |  | |
| Age† | n at-risk (no· of cases) |  |  |  |  | |
| 19-24 | 218,141 (769) | 0·35 (0·33-0·38) | 0·29 (0·26-0·32) | 0·29 (0·20-0·39) | 0·45 (0·41-0·50) | |
| 25-29 | 171,060 (2,908) | 1·70 (1·64-1·76) | 1·46 (1·37-1·54) | 1·60 (1·33-1·88) | 1·92 (1·83-2·01) | |
| 30-34 | 152,069 (5,288) | 3·48 (3·39-3·57) | 2·31 (2·14-2·49) | 2·50 (2·28-2·71) | 4·00 (3·88-4·12) | |
| Overall | 541,270 (8,965) | 1·66 (1·62-1·69) | 0·94 (0·90-0·97) | 1·67 (1·54-1·79) | 2·27 (2·21-2·32) | |
| **Cervical cancer** | |  |  |  |  | |
| Age† | n at-risk (no· of cases) |  |  |  |  | |
| 19-24 | 218,141 (33) | 0·02 (0·01-0·02) | 0·02 (0·01-0·02) | 0·02 (0·00-0·05) | 0·01 (0·01-0·02) | |
| 25-29 | 171,060 (172) | 0·10 (0·09-0·12) | 0·08 (0·06-0·10) | 0·08 (0·02-0·14) | 0·12 (0·10-0·15) | |
| 30-34 | 152,069 (464) | 0·31 (0·28-0·33) | 0·18 (0·13-0·23) | 0·19 (0·13-0·25) | 0·36 (0·33-0·40) | |
| Overall | 541,270 (669) | 0·12 (0·11-0·13) | 0·06 (0·05-0·07) | 0·12 (0·09-0·15) | 0·18 (0·16-0·20) | |

* Two or more doses was defined as at least two doses of HPV vaccine received, irrespective of the interval between the doses.

** One dose was defined as receiving one dose of the HPV vaccine.

*** Unvaccinated persons were those who had never received a dose of any HPV vaccine.

n: number of females

\# For the calculation of the proportions, the population was limited to those who contacted a doctor in 2024.

† Participants were grouped according to their age at the end of follow-up in 2024; outcomes were counted irrespective of the age at diagnosis.

# **Supplementary Table S3: Crude and adjusted Cox proportional hazards models on the association of HPV vaccination status (two or more doses, one dose, unvaccinated) and other factors on the risk of diagnosis of precancerous lesions and invasive cervical cancer in females (n=669,053)**

| **Covariable** | **Precancerous lesions  Hazard ratio (95% confidence interval)** | **Cervical cancer  Hazard ratio (95% confidence interval)** |
| --- | --- | --- |
| **Crude Model (Model 5)$** |  |  |
| **HPV vaccination status** |  |  |
| One Dose** (Ref. Unvaccinated) | 0·79 (0·75-0·84) | 0·66 (0·52-0·83) |
| Two or more doses*  (Ref. Unvaccinated***) | 0·70 (0·66-0·73) | 0·53 (0·44-0·64) |
| **Fully Adjusted Model (Model 6)§** |  |  |
| **HPV vaccination status** |  |  |
| One Dose (Ref. Unvaccinated) | 0·68 (0·64-0·73) | 0·50 (0·38-0·66) |
| Two or more doses  (Ref. Unvaccinated) | 0·60 (0·57-0·64) | 0·45 (0·34-0·57) |
| **Cervical Cancer Screening** |  |  |
| Yes (Ref. No) | 6·97 (6·27-7·75) | 4·85 (3·28-7·17) |
| **Age at vaccination** |  |  |
| 9-14 years (Ref. ≥15 years) | 1·17 (1·08-1·27) | 1·04 (0·74-1·46) |
| **Contraception before HPV vaccination** | |  |
| Yes (Ref. No) | 1·38 (1·27-1·49) | 1·95 (1·42-2·67) |
| **Place of residence+** |  |  |
| Larger cities (Ref. Rural areas) | 1·24 (1·18-1·30) | 1·27 (1·06-1·52) |

$ Model 5 is the crude model, includes all females, and evaluates the association between HPV vaccination status and the risk of diagnosis.

§ Model 6 is the fully adjusted model and includes all females, assesses the association between HPV vaccination status and the risk of diagnosis, and adjusts for cervical cancer screening participation, age at vaccination, place of residence, and contraceptive prescription prior to vaccination.

* Two or more doses was defined as at least two doses of HPV vaccine received, irrespective of the interval between the doses.

** One dose was defined as receiving one dose of the HPV vaccine.

*** Unvaccinated females were those who had never received a dose of any HPV vaccine.

+ Place of residence: larger cities (district-free larger cities) versus rural areas (district cities, rural districts, and sparsely populated rural districts) in Bavaria.

Ref.: Reference

# **Supplementary Table S4: Adjusted Cox proportional hazards models on the association of HPV vaccination status (two or more doses, one dose, unvaccinated) and other factors on the risk of diagnosis of precancerous lesions and invasive cervical cancer in females (Model 7: n=669,053, Model 8: n=432,530).**

| Covariable | **Precancerous lesions  Hazard ratio (95% confidence interval)** | **Cervical cancer  Hazard ratio (95% confidence interval)** |
| --- | --- | --- |
| **Secondary analysis (Model 7)$** |  |  |
| **HPV vaccination status** |  |  |
| One Dose** (Ref. Unvaccinated) | 0·69 (0·63-0·73) | 0·51 (0·38-0·68) |
| Two or more doses* (Ref. Unvaccinated***) | 0·61 (0·57-0·65) | 0·46 (0·35-0·59) |
| **Cervical Cancer Screening** |  |  |
| Yes (Ref. No) | 6·97 (6·27-7·75) | 4·86 (3·29-7·18) |
| **Age at vaccination** |  |  |
| 9-14 years (Ref. ≥15 years) | 1·17 (1·08-1·27) | 1·04 (0·74-1·47) |
| **Contraception before HPV vaccination** | |  |
| Yes (Ref. No) | 1·38 (1·27-1·49) | 1·95 (1·42-2·67) |
| **Place of residence** |  |  |
| Larger cities (Ref. Rural areas) | 1·24 (1·18-1·30) | 1·27 (1·06–1·53) |
| **Vaccine Type** |  |  |
| Cervarix® (Ref. Gardasil® / Gardasil 9®) | 0·87 (0·76-1·00) | 0·69 (0·36-1·30) |
| **Secondary analysis (Model 8)§** |  |  |
| **HPV vaccination status** |  |  |
| One Dose (Ref. Unvaccinated) | 0·88 (0·79-0·98) | 0·78 (0·48-1·28) |
| Two or more doses (Ref. Unvaccinated) | 0·63 (0·58-0·70) | 0·55 (0·36-0·86) |
| **Cervical Cancer Screening** |  |  |
| Yes (Ref. No) | 7·28 (6·17-8·60) | 6·29 (2·91-13·57) |
| **Age at vaccination** |  |  |
| 9-14 years (Ref. ≥15 years) | 1·17 (1·05-1·30) | 1·24 (0·76-2·02) |
| **Contraception before HPV vaccination** | |  |
| Yes (Ref. No) | 1·57 (1·36-1·81) | 1·21 (0·58-2·52) |
| **Place of residence+** |  |  |
| Larger cities (Ref. Rural areas) | 1·28 (1·17-1·39) | 1·33 (0·93-1·91) |
| **J1 Examination** |  |  |
| Yes (Ref. No) | 0·93 (0·87-1·00) | 1·14 (0·84-1·56) |

$ Model 7 includes all females, assesses the association between HPV vaccination status and the risk of diagnosis, and adjusts for cervical cancer screening participation, age at vaccination, place of residence, and contraceptive prescription prior to vaccination, as well as for vaccine type.

§ Model 8 is restricted to cohorts born between 1996 and 2005 and additionally adjusts for J1 examination status.

* Two or more doses was defined as at least two doses of HPV vaccine received, irrespective of the interval between the doses.

** One dose was defined as receiving one dose of the HPV vaccine.

*** Unvaccinated persons were those who had never received a dose of any HPV vaccine.

+ Place of residence: larger cities (district-free larger cities) versus rural areas (district cities, rural districts, and sparsely populated rural districts) in Bavaria.

Ref.: Reference

# **Supplementary Table S5: Adjusted Cox proportional hazards models on the association of HPV vaccination status (fully vaccinated, partially vaccinated, unvaccinated) and other factors on the risk of diagnosis of precancerous lesions and invasive cervical cancer in females (Model 3: n= 669,053, Model 4: n=432,530)**

| **Covariable** | **Precancerous lesions  Hazard ratio (95% confidence interval)** | **Cervical cancer  Hazard ratio (95% confidence interval)** |
| --- | --- | --- |
| **Secondary analysis (Model 3)$** |  |  |
| **HPV vaccination status** |  |  |
| Partially Vaccinated**  (Ref. Unvaccinated) | 0·53 (0·49-0·58) | 0·41 (0·30-0·57) |
| Fully vaccinated*  (Ref. Unvaccinated***) | 0·49 (0·45-0·53) | 0·36 (0·26-0·49) |
| **Cervical Cancer Screening** |  |  |
| Yes (Ref. No) | 6·97 (6·27-7·75) | 4·86 (3·29-7·19) |
| **Age at vaccination** |  |  |
| Up to 14 years (Ref. ≥15 years) | 1·34 (1·24-1·46) | 1·16 (0·81-1·67) |
| **Contraception before HPV vaccination** | |  |
| Yes (Ref. No) | 1·58 (1·45-1·73) | 2·13 (1·51-3·01) |
| **Place of residence** |  |  |
| Larger cities (Ref. Rural areas) | 1·24 (1·18-1·31) | 1·28 (1·06-1·53) |
| **Vaccine Type** |  |  |
| Cervarix®  (Ref. Gardasil® / Gardasil 9®) | 0·86 (0·75-0·98) | 0·65 (0·35-1·24) |
| **Secondary Analysis (Model 4)§** |  |  |
| **HPV vaccination status** |  |  |
| Partially Vaccinated  (Ref. Unvaccinated) | 0·66 (0·57-0·76) | 0·60 (0·32-1·12) |
| Fully Vaccinated  (Ref. Unvaccinated) | 0·55 (0·49-0·62) | 0·56 (0·34-0·93) |
| **Screening** |  |  |
| Yes (Ref. No) | 7·30 (6·18-8·62) | 6·29 (2·91-13·57) |
| **Age at vaccination** |  |  |
| 9-14 years (Ref. ≥15 years) | 1·27 (1·13-1·43) | 1·16 (0·69-1·96) |
| **Contraception before HPV vaccination** | |  |
| Yes (Ref. No) | 1·66 (1·43-1·93) | 1·05 (0·48-2·30) |
| **Place of residence+** |  |  |
| Larger cities (Ref. Rural areas) | 1·28 (1·18-1·40) | 1·34 (0·93-1·92) |
| **J1 Examination** |  |  |
| Yes (Ref. No) | 0·94 (0·87-1·01) | 1·14 (0·84-1·56) |

$ Model 3 includes all females, assesses the association between HPV vaccination status and the risk of diagnosis and adjusts for cervical cancer screening participation, age at vaccination, place of residence, and contraceptive prescription prior to vaccination, as well as for vaccine type.

§ Model 4 is restricted to cohorts born between 1996 and 2005 and additionally adjusts for J1 examination status.

* Fully vaccinated was defined as two doses of vaccine received at least 5 months apart for the 9-14 group and three doses received from age 15 years, irrespective of the interval between the doses.

** Any deviation from the fully vaccinated definition was considered as partially vaccinated.

*** Unvaccinated females were those who had never received a dose of any HPV vaccine.

+ Place of residence: larger cities (district-free larger cities) versus rural areas (district cities, rural districts, and sparsely populated rural districts) in Bavaria.

Ref.: Reference

# **Supplementary Table S6: Cumulative incidence estimates of precancerous lesions and invasive cervical cancer, stratified by vaccination status**

| **Cumulative Incidence (95% Confidence Interval)** | | | | | |
| --- | --- | --- | --- | --- | --- |
| **Age** | **Fully vaccinated*** | **Partially vaccinated**** | **Unvaccinated***** | **Two or more doses#** | **One dose##** |
| **Precancerous Cervical Lesions** | | | | | |
| 18 | 0·000 (0·000–0·000) | 0·000 (0·000–0·000) | 0·000 (0·000–0·000) | 0·000 (0·000–0·000) | 0·000 (0·000–0·000) |
| 19 | 0·001 (0·000–0·001) | 0·001 (0·001–0·001) | 0·001 (0·001–0·001) | 0·001 (0·001–0·001) | 0·001 (0·000–0·001) |
| 20 | 0·001 (0·001–0·002) | 0·002 (0·002–0·002) | 0·002 (0·002–0·002) | 0·001 (0·001–0·002) | 0·002 (0·001–0·002) |
| 21 | 0·002 (0·002–0·002) | 0·003 (0·003–0·003) | 0·003 (0·003–0·004) | 0·002 (0·002–0·003) | 0·003 (0·002–0·003) |
| 22 | 0·004 (0·003–0·004) | 0·005 (0·004–0·005) | 0·005 (0·005–0·006) | 0·004 (0·004–0·004) | 0·004 (0·004–0·005) |
| 23 | 0·005 (0·005–0·005) | 0·006 (0·006–0·007) | 0·007 (0·007–0·007) | 0·005 (0·005–0·006) | 0·006 (0·006–0·007) |
| 24 | 0·006 (0·006–0·007) | 0·008 (0·007–0·009) | 0·009 (0·009–0·010) | 0·007 (0·006–0·007) | 0·008 (0·007–0·009) |
| 25 | 0·008 (0·007–0·008) | 0·010 (0·009–0·011) | 0·012 (0·012–0·013) | 0·008 (0·008–0·009) | 0·010 (0·009–0·011) |
| 26 | 0·010 (0·009–0·010) | 0·012 (0·011–0·013) | 0·015 (0·015–0·016) | 0·010 (0·010–0·011) | 0·012 (0·011–0·013) |
| 27 | 0·012 (0·011–0·012) | 0·014 (0·013–0·015) | 0·018 (0·018–0·019) | 0·012 (0·011–0·012) | 0·014 (0·013–0·015) |
| 28 | 0·014 (0·013–0·014) | 0·016 (0·015–0·017) | 0·021 (0·021–0·022) | 0·014 (0·013–0·015) | 0·016 (0·014–0·017) |
| 29 | 0·015 (0·014–0·016) | 0·018 (0·017–0·019) | 0·025 (0·024–0·025) | 0·016 (0·015–0·017) | 0·018 (0·016–0·019) |
| 30 | 0·017 (0·016–0·018) | 0·019 (0·018–0·020) | 0·028 (0·027–0·029) | 0·017 (0·016–0·018) | 0·019 (0·017–0·020) |
| 31 | 0·019 (0·017–0·020) | 0·021 (0·019–0·022) | 0·032 (0·031–0·033) | 0·019 (0·018–0·020) | 0·020 (0·019–0·022) |
| 32 | 0·020 (0·019–0·022) | 0·023 (0·021–0·024) | 0·035 (0·034–0·036) | 0·021 (0·020–0·022) | 0·022 (0·021–0·024) |
| 33 | 0·022 (0·020–0·025) | 0·024 (0·023–0·026) | 0·038 (0·037–0·039) | 0·023 (0·021–0·025) | 0·024 (0·022–0·026) |
| 34 | 0·025 (0·020–0·029) | 0·025 (0·024–0·027) | 0·041 (0·040–0·043) | 0·024 (0·022–0·027) | 0·025 (0·023–0·027) |
| **Invasive Cervical Cancer** | | | | | |
| 18 | 0·000 (0·000–0·000) | 0·000 (0·000–0·000) | 0·000 (0·000–0·000) | 0·000 (0·000–0·000) | 0·000 (0·000–0·000) |
| 19 | 0·000 (0·000–0·000) | 0·000 (0·000–0·000) | 0·000 (0·000–0·000) | 0·000 (0·000–0·000) | 0·000 (0·000–0·000) |
| 20 | 0·000 (0·000–0·000) | 0·000 (0·000–0·000) | 0·000 (0·000–0·000) | 0·000 (0·000–0·000) | 0·000 (0·000–0·000) |
| 21 | 0·000 (0·000–0·000) | 0·000 (0·000–0·000) | 0·000 (0·000–0·000) | 0·000 (0·000–0·000) | 0·000 (0·000–0·000) |
| 22 | 0·000 (0·000–0·000) | 0·000 (0·000–0·000) | 0·000 (0·000–0·000) | 0·000 (0·000–0·000) | 0·000 (0·000–0·000) |
| 23 | 0·000 (0·000–0·000) | 0·000 (0·000–0·000) | 0·000 (0·000–0·001) | 0·000 (0·000–0·000) | 0·000 (0·000–0·001) |
| 24 | 0·000 (0·000–0·000) | 0·000 (0·000–0·001) | 0·001 (0·001–0·001) | 0·000 (0·000–0·000) | 0·000 (0·000–0·001) |
| 25 | 0·000 (0·000–0·000) | 0·001 (0·000–0·001) | 0·001 (0·001–0·001) | 0·000 (0·000–0·001) | 0·001 (0·000–0·001) |
| 26 | 0·000 (0·000–0·001) | 0·001 (0·000–0·001) | 0·001 (0·001–0·001) | 0·000 (0·000–0·001) | 0·001 (0·000–0·001) |
| 27 | 0·001 (0·000–0·001) | 0·001 (0·001–0·001) | 0·001 (0·001–0·001) | 0·001 (0·001–0·001) | 0·001 (0·001–0·001) |
| 28 | 0·001 (0·001–0·001) | 0·001 (0·001–0·001) | 0·002 (0·001–0·002) | 0·001 (0·001–0·001) | 0·001 (0·001–0·001) |
| 29 | 0·001 (0·001–0·001) | 0·001 (0·001–0·001) | 0·002 (0·002–0·002) | 0·001 (0·001–0·001) | 0·001 (0·001–0·002) |
| 30 | 0·001 (0·001–0·001) | 0·001 (0·001–0·002) | 0·002 (0·002–0·002) | 0·001 (0·001–0·001) | 0·001 (0·001–0·002) |
| 31 | 0·001 (0·001–0·001) | 0·001 (0·001–0·002) | 0·003 (0·002–0·003) | 0·001 (0·001–0·001) | 0·001 (0·001–0·002) |
| 32 | 0·001 (0·001–0·002) | 0·002 (0·001–0·002) | 0·003 (0·003–0·003) | 0·001 (0·001–0·002) | 0·002 (0·001–0·002) |
| 33 | 0·001 (0·001–0·002) | 0·002 (0·001–0·002) | 0·003 (0·003–0·004) | 0·002 (0·001–0·002) | 0·002 (0·001–0·002) |
| 34 | 0·001 (0·001–0·002) | 0·002 (0·001–0·002) | 0·004 (0·003–0·004) | 0·002 (0·001–0·002) | 0·002 (0·001–0·002) |

* Fully vaccinated was defined as two doses of vaccine received at least 5 months apart for the 9-14 age group and three doses received from age 15 years, irrespective of the interval between the doses.

** Any deviation from the fully vaccinated definition was considered as partially vaccinated.

*** Unvaccinated females were those who had never received a dose of any HPV vaccine.

\# Two or more doses was defined as at least two doses of HPV vaccine received, irrespective of the interval between the doses.

\## One dose was defined as receiving one dose of the HPV vaccine

# **Supplementary Table S7: Cumulative incidence estimates of precancerous lesions and invasive cervical cancer, stratified by age at vaccination and contraceptive prescription status, in vaccinated cohorts only**

| **Cumulative Incidence (95% Confidence Interval)** | | | | |
| --- | --- | --- | --- | --- |
| **Age** | **9-14 years** | **≥15 years** | **No contraception before HPV vaccination** | **Contraception before HPV vaccination** |
| **Precancerous Cervical Lesions** | | | | |
| 18 | 0·000 (0·000–0·000) | 0·000 (0·000–0·000) | 0·000 (0·000–0·000) | 0·000 (0·000–0·000) |
| 19 | 0·001 (0·001–0·001) | 0·001 (0·000–0·001) | 0·001 (0·000–0·001) | 0·001 (0·001–0·001) |
| 20 | 0·002 (0·001–0·002) | 0·001 (0·001–0·002) | 0·001 (0·001–0·002) | 0·002 (0·002–0·002) |
| 21 | 0·002 (0·002–0·003) | 0·003 (0·002–0·003) | 0·002 (0·002–0·002) | 0·004 (0·003–0·004) |
| 22 | 0·004 (0·004–0·004) | 0·004 (0·004–0·004) | 0·004 (0·003–0·004) | 0·006 (0·005–0·006) |
| 23 | 0·006 (0·005–0·006) | 0·005 (0·005–0·006) | 0·005 (0·005–0·005) | 0·008 (0·007–0·009) |
| 24 | 0·007 (0·006–0·008) | 0·007 (0·006–0·007) | 0·006 (0·006–0·006) | 0·011 (0·010–0·012) |
| 25 | 0·009 (0·008–0·009) | 0·008 (0·008–0·009) | 0·008 (0·007–0·008) | 0·013 (0·012–0·014) |
| 26 | 0·011 (0·010–0·012) | 0·010 (0·010–0·011) | 0·009 (0·009–0·010) | 0·016 (0·015–0·017) |
| 27 | 0·013 (0·012–0·014) | 0·012 (0·011–0·013) | 0·011 (0·010–0·011) | 0·018 (0·017–0·019) |
| 28 | 0·015 (0·014–0·016) | 0·014 (0·013–0·015) | 0·013 (0·012–0·013) | 0·020 (0·019–0·022) |
| 29 | 0·017 (0·016–0·018) | 0·016 (0·015–0·016) | 0·014 (0·014–0·015) | 0·022 (0·021–0·024) |
| 30 | 0·018 (0·017–0·019) | 0·017 (0·016–0·018) | 0·015 (0·015–0·016) | 0·024 (0·022–0·026) |
| 31 | 0·020 (0·017–0·023) | 0·019 (0·018–0·020) | 0·017 (0·016–0·018) | 0·026 (0·024–0·028) |
| 32 | - | 0·021 (0·020–0·022) | 0·019 (0·018–0·020) | 0·028 (0·026–0·030) |
| 33 | - | 0·022 (0·021–0·024) | 0·020 (0·019–0·022) | 0·030 (0·028–0·032) |
| 34 | - | 0·024 (0·022–0·025) | 0·021 (0·019–0·023) | 0·031 (0·029–0·034) |
| **Invasive Cervical Cancer** | | | | |
| 18 | 0·000 (0·000–0·000) | 0·000 (0·000–0·000) | 0·000 (0·000–0·000) | 0·000 (0·000–0·000) |
| 19 | 0·000 (0·000–0·000) | 0·000 (0·000–0·000) | 0·000 (0·000–0·000) | 0·000 (0·000–0·000) |
| 20 | 0·000 (0·000–0·000) | 0·000 (0·000–0·000) | 0·000 (0·000–0·000) | 0·000 (0·000–0·000) |
| 21 | 0·000 (0·000–0·000) | 0·000 (0·000–0·000) | 0·000 (0·000–0·000) | 0·000 (0·000–0·000) |
| 22 | 0·000 (0·000–0·000) | 0·000 (0·000–0·000) | 0·000 (0·000–0·000) | 0·000 (0·000–0·000) |
| 23 | 0·000 (0·000–0·000) | 0·000 (0·000–0·000) | 0·000 (0·000–0·000) | 0·000 (0·000–0·001) |
| 24 | 0·000 (0·000–0·000) | 0·000 (0·000–0·000) | 0·000 (0·000–0·000) | 0·000 (0·000–0·001) |
| 25 | 0·000 (0·000–0·001) | 0·000 (0·000–0·001) | 0·000 (0·000–0·000) | 0·001 (0·000–0·001) |
| 26 | 0·001 (0·000–0·001) | 0·001 (0·000–0·001) | 0·000 (0·000–0·001) | 0·001 (0·001–0·001) |
| 27 | 0·001 (0·000–0·001) | 0·001 (0·001–0·001) | 0·001 (0·000–0·001) | 0·001 (0·001–0·002) |
| 28 | 0·001 (0·001–0·001) | 0·001 (0·001–0·001) | 0·001 (0·000–0·001) | 0·002 (0·001–0·002) |
| 29 | 0·001 (0·001–0·001) | 0·001 (0·001–0·001) | 0·001 (0·001–0·001) | 0·002 (0·001–0·002) |
| 30 | 0·001 (0·001–0·001) | 0·001 (0·001–0·001) | 0·001 (0·001–0·001) | 0·002 (0·002–0·003) |
| 31 | 0·001 (0·001–0·001) | 0·001 (0·001–0·002) | 0·001 (0·001–0·001) | 0·002 (0·002–0·003) |
| 32 |  | 0·002 (0·001–0·002) | 0·001 (0·001–0·001) | 0·002 (0·002–0·003) |
| 33 |  | 0·002 (0·001–0·002) | 0·001 (0·001–0·002) | 0·003 (0·002–0·003) |
| 34 |  | 0·002 (0·001–0·002) | 0·001 (0·001–0·002) | 0·003 (0·002–0·003) |

a

**Cumulative Incidence**

**Age at follow-up (years)**

**Age at follow-up (years)**

b

**Cumulative Incidence**

**Age at follow-up (years)**

**Age at follow-up (years)**

# **Supplementary Figure S1: Cumulative incidence of a) Precancerous lesions (ICD-10 N87·1, N87·2, D06) and b) Cervical cancer (ICD-10 C53) by age at follow-up stratified by vaccination status in females from 9-34 years in Bavaria (n=669,053).**

Definition: 2+ dose was defined as two or more doses of HPV vaccine received, irrespective of the interval between the doses; 1 dose was defined as receiving one dose of the HPV vaccine. Unvaccinated females were those who had never received a dose of any HPV vaccine.
